# *Lactiplantibacillus plantarum* as a novel platform for production and purification of integral membrane proteins using RseP as the benchmark

**DOI:** 10.1038/s41598-023-41559-7

**Published:** 2023-09-01

**Authors:** Sofie S. Kristensen, Marie V. Lukassen, Suzana Siebenhaar, Dzung B. Diep, J. Preben Morth, Geir Mathiesen

**Affiliations:** 1 Faculty of Chemistry, Biotechnology and Food Science, Norwegian University of Life Sciences (NMBU), Ås, Norway; 2 Department of Biotechnology and Biomedicine, Technical University of Denmark (DTU), Kongens Lyngby, Denmark

**Keywords:** Isolation, separation and purification, Chromatography, Protein purification, Biochemistry, Biological techniques, Biotechnology, Chemical biology, Microbiology, Molecular biology, Structural biology

## Abstract

The present study describes a detailed procedure for expressing and purifying the integral membrane protein RseP using the pSIP system and *Lactiplantibacillus plantarum* as an expression host. RseP is a membrane-bound site-2-protease and a known antibacterial target in multiple human pathogens. In the present study, we screened five RseP orthologs from Gram-positive bacteria and found RseP from *Enterococcus faecium* (EfmRseP) to yield the highest protein levels. The production conditions were optimized and EfmRseP was purified by immobilized metal ion affinity chromatography followed by size-exclusion chromatography. The purification resulted in an overall yield of approximately 1 mg of pure protein per 3 g of wet-weight cell pellet. The structural integrity of the purified protein was confirmed using circular dichroism. We further assessed the expression and purification of RseP from *E. faecium* in the Gram-negative *Escherichia coli*. Detection of soluble protein failed in two of the three *E. coli* strains tested. Purification of EfmRseP expressed in *E. coli* C43(DE3) resulted in a protein with lower purity compared to EfmRseP expressed in *L. plantarum*. To our knowledge, this is the first time *L. plantarum* and the pSIP expression system have been applied for the production of membrane proteins.

## Introduction

Integral membrane proteins (IMPs) reside within the lipid membrane surrounding cells and organelles and play a pivotal role in multiple cellular processes, including nutrient transport, molecular recognition, and maintenance of cell integrity. It is estimated that membrane proteins account for 20–30% of the proteome in most organisms and 60% of all drug targets, thereby highlighting the importance of membrane proteins in the cell^1–4^.

**Figure 1:**
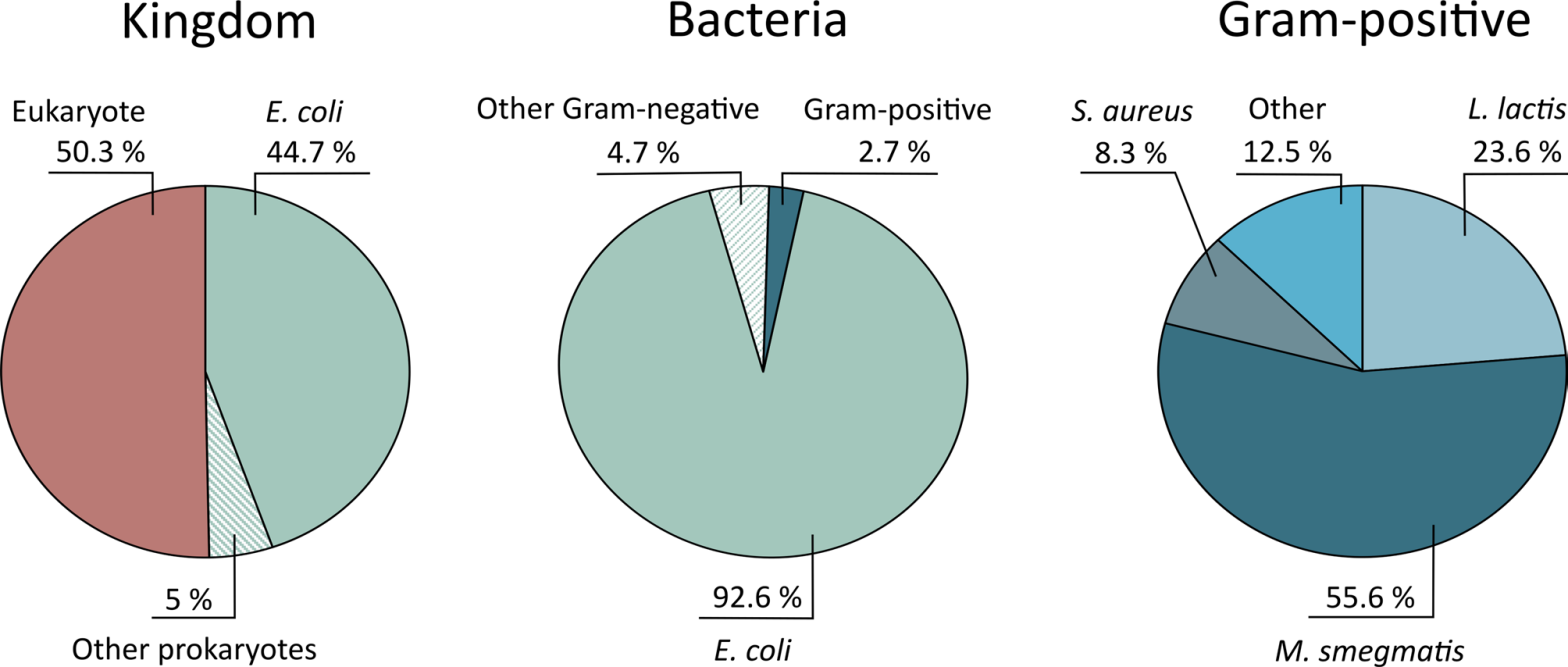
Comparison of the expression hosts used for the production of transmembrane proteins used in structural studies. The percentage of the entries in the Protein Data Bank annotated as transmembrane proteins distributed according to expression host.

*Escherichia coli* is the most widely used expression host for recombinant expression of IMPs (Fig. 1); however, many membrane proteins fail to fold or get trafficked to the *E. coli* membrane correctly*,* resulting in the formation of inclusion bodies^5,6^. Several factors may contribute to insolubility and protein misfolding in *E. coli*, including lack of specific chaperones, mismatch in codon usage and differences in membrane lipid composition. Several alternative expression systems have been developed to circumvent these limitations, with the central principle being that protein expression may be more successful in a host organism more closely related to the organism from which the protein of interest is derived (Fig. 1)^7,8^.

Atomic structures of IMPs are generally underrepresented in the Protein Data Bank (https://www.rcsb.org/), only accounting for approximately 3.75% of the total entries (per. November 2022). Various technical challenges impede the structural characterization of IMPs, with obtaining sufficient amounts of pure and homogeneous protein generally being the first major bottleneck. Among Gram-positive expression hosts used for protein production to generate IMP structures in the Protein Data Bank, *Mycobacterium smegmatis* and *Lactococcus lactis* are the most commonly used (Fig. 1). *M. smegmatis* has been developed as an alternative expression host for mycobacterial proteins, as the expression of proteins from mycobacterial species, such as *Mycobacterium tuberculosis*, are particularly challenging in *E. coli*^9,10^. *M. smegmatis* has so far not been extensively used for the expression of proteins from non-mycobacterial species. In contrast, *L. lactis* has been applied in the production of both eukaryotic and prokaryotic proteins, highlighting the versatility of this expression system^11–14^.

The use of *L. lactis* for membrane protein production has several advantages, including (i) a small genome with limited proteolytic activity; (ii) the monoderm cell envelope; (iii) a well-developed genetic tool-box; and (iv) the ability to reach high cell densities without aeration, thereby allowing expression at industrial scale using bioreactors^14–18^. Several inducible and constitutive expression system has been developed for use in *L. lactis*, with the nisin-controlled expression system (NICE) being the most extensively used^19^. Various membrane proteins have successfully been structurally characterized using the NICE system in *L. lactis*^20–24^ (Fig. 1).

Other lactic acid bacteria, such as *Lactiplantibacillus plantarum*, also show significant potential for the overproduction of heterologous proteins^25,26^. Several inducible and constitutive expression systems are compatible with lactobacillal *s*pecies, including the pheromone inducible pSIP vectors, which have been extensively used for the expression, secretion, and surface anchoring of a range of heterologous proteins^25,27–31^. The pSIP system is based on the promotors and regulatory genes involved in the production of the class-II bacteriocin sakacin P in *Latilactobacillus sakei.* The pSIP expression system offers tight regulation of gene expression. The promotor upstream of the target gene is strictly controlled by the two-component system *sppKR*, which depends on the external addition of the peptide pheromone SppIP for induction^27,28^.

While intracellular production of heterologous proteins is well established in *L. plantarum*, expression of IMPs has not yet been evaluated. In the present study, we explore the potential of the pSIP-expression system in *L. plantarum* as a novel platform for the production and purification of the site-2-protease RseP. RseP is a membrane-embedded metalloprotease involved in regulated intramembrane proteolysis (RIP), which is a widely distributed signaling mechanism used by bacteria to control a diverse array of cellular processes, including stress response, nutrient uptake, and virulence^32–36^. Due to its prominent role in bacterial physiology and virulence, RseP is considered an attractive antimicrobial target^37,38^. RseP of selected Gram-positive bacteria acts as a receptor for a specific group of bacteriocins known as the LsbB-family, further highlighting RseP as an potential antimicrobial target^39–43^. Notably, the bacteriocin enterocin K1 (EntK1), which is highly potent against vancomycin resistant bacteria, depends on interaction with enterococcal RseP for its antimicrobial activity^40,43,44^. The pSIP system has recently been exploited to perform mutational analysis of RseP, revealing molecular details on the interaction between EntK1 and RseP from *Enterococcus faecium*^43^. This knowledge, in combination with further structural characterization of RseP, may provide a powerful basis for the rational design of novel bacteriocins. Thus, in this study, we express and purify RseP derived from the Gram-positive *E. faecium* using the pSIP system and *L. plantarum* as the expression host. To our knowledge, this is the first time *L. plantarum* and the pSIP expression system have been applied for the production of membrane proteins.

## Results

### Identification of an RseP ortholog for expression and purification

**Figure 2:**
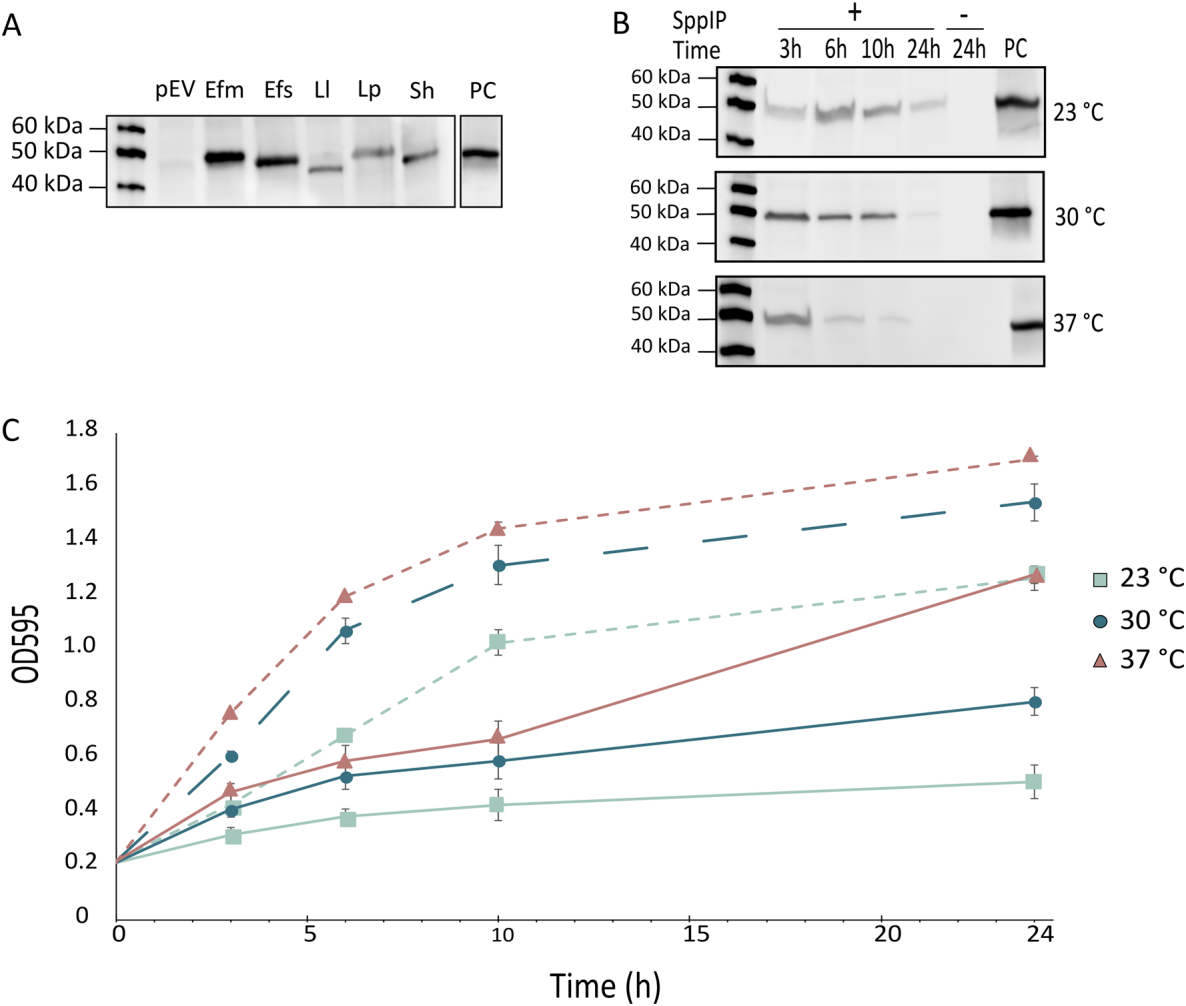
Optimalization of RseP protein production. (**A**) Heterologous production of RseP derived from five gram-positive bacteria was evaluated in *L. plantarum* WCFS1 using western blot analysis: *E. faecium* (Efm), *E. faecalis* (Efs), *L. lactis* (Ll), *L. plantarum* (Lp) and *S. haemolyticus* (Sh). The production was evaluated at 37 °C and induced with 25 ng/ml and harvested after 3 h. The empty vector (pEV) was used as a negative control. (**B**) Small-scale expression screening of EfmRseP was conducted in 50 ml cultures at three temperatures: 23 °C, 30 °C and 37 °C, and harvested at various time points after induction: 3-, 6-, 10- and 24 h. All cultures were induced with 25 ng/ml SppIP at OD_600_ ~ 0.3, except a non-induced culture (–) harvested 24 h after induction which was used as a negative control for each temperature. In the western blots (**A**,**B**), a purified 6xHis-protein was used as a positive control (PC). The original full-length blots are presented in supplementary Fig. S6. (**C**) Growth curve of *L. plantarum* harboring pEfmRseP induced (25 ng/ml SppIP; solid line) and non-induced samples (dashed line) at 23 °C, 30 °C and 37 °C. OD_595_ was measured at the time of induction (0 h), and after 3-, 6-, 10- and 24 h post induction. The experiment was performed using three biological replicates. The standard deviation is indicated at each data point.

Small changes in the amino acid sequence or length of heterologously expressed proteins may affect expression levels and solubility^45–47^. Orthologue screening has proven to be a successful approach for identifying protein variants which can be produced with high yields. Thus, expression levels and functionality of five different RseP orthologs were evaluated using the pSIP-expression system. To screen for functional expression of RseP, the sensitivity of all recombinant strains were challenged against the EntK1 of the LsbB bacteriocin family in a spot-on-lawn assay (Fig. S1). All orthologs screened, except for RseP from *L. plantarum* (LpRseP), act as a receptor for bacteriocins of the LsbB family in their native organism^39–41^. LpRseP was included in the expression screen as we hypothesized that the expression of a protein native to the expression host might ease the expression burden, thus resulting in higher protein production. As expected, all recombinant strains except *L. plantarum* expressing LpRseP, were sensitive to the LsbB family bacteriocin EntK1, suggesting that the RseP orthologs are functionally produced in vivo (Fig. S1). A C-terminal 6xHis-tag was included in all constructs to facilitate purification and detection of RseP. Western blot analysis confirmed the production of all recombinant RseP orthologs in *L. plantarum* (Fig. 2A). However, the expression levels varied considerably. Surprisingly, RseP derived from the host (LpRseP) and the closely related *L. lactis* (LlRseP) resulted in the lowest production levels, while RseP derived from *Enterococcus faecium* (EfmRseP) yielded the highest level. Thus, further optimization of protein production and purification was performed using the EfmRseP construct only.

### Small-scale optimization of protein production in *L. plantarum*

To identify optimal conditions for RseP production in *L. plantarum*, 50 ml growth cultures were used to study the effects of various incubation temperatures, inducer concentrations, and harvesting time on the protein production. Initially, the production was analyzed at three different temperatures: 23 °C, 30 °C and 37 °C, and harvested after 3-, 6-, 10- or 24 h. The volume of protein extract applied in the analyzed was normalized based on OD_600_ at the harvesting time point. Western blot analysis showed that the production level was largely temperature-dependent (Fig. 2). A considerable level of EfmRseP was detected from 3- to 10 h after induction at 30 °C and 23 °C, while the amount decreased significantly over the same period of time at 37 °C. Although the normalized protein production was comparable at 3- and 10 h after induction at 30 °C, the total protein yield was estimated as higher after 10 h due to an increase in biomass (Fig. 2B). A prolonged incubation past 10 h resulted in less EfmRseP regardless of the incubation temperature, which is not ideal when the goal is to produce as much EfmRseP as possible.

Induction of the protein expression significantly affected the cell growth of *L. plantarum* expressing EfmRseP at all temperatures (Fig. 2C). Notably, the lack of protein expression after 10 h at 37 °C correlates with the rapid increase in growth rate observed at the same time point (Fig. 2B,C), suggesting that induction has ceased. Varying the inducer concentration (12.5–50 ng/ml) did not prolong EfmRseP production time nor affect the growth rate (Fig. S2).

*L. plantarum* is a facultative anaerobe organism, thus aeration is not recommended for optimal growth. However, enhanced flow of nutrients might be advantageous for prolonged incubations. Thus, growth and protein expression were monitored in cultures with minimal agitation (200 rpm). However, we did not observe any significant effect on neither growth rate nor protein production levels (data not shown).

### Large-scale purification of recombinant EfmRseP from *L. plantarum*

**Figure 3:**
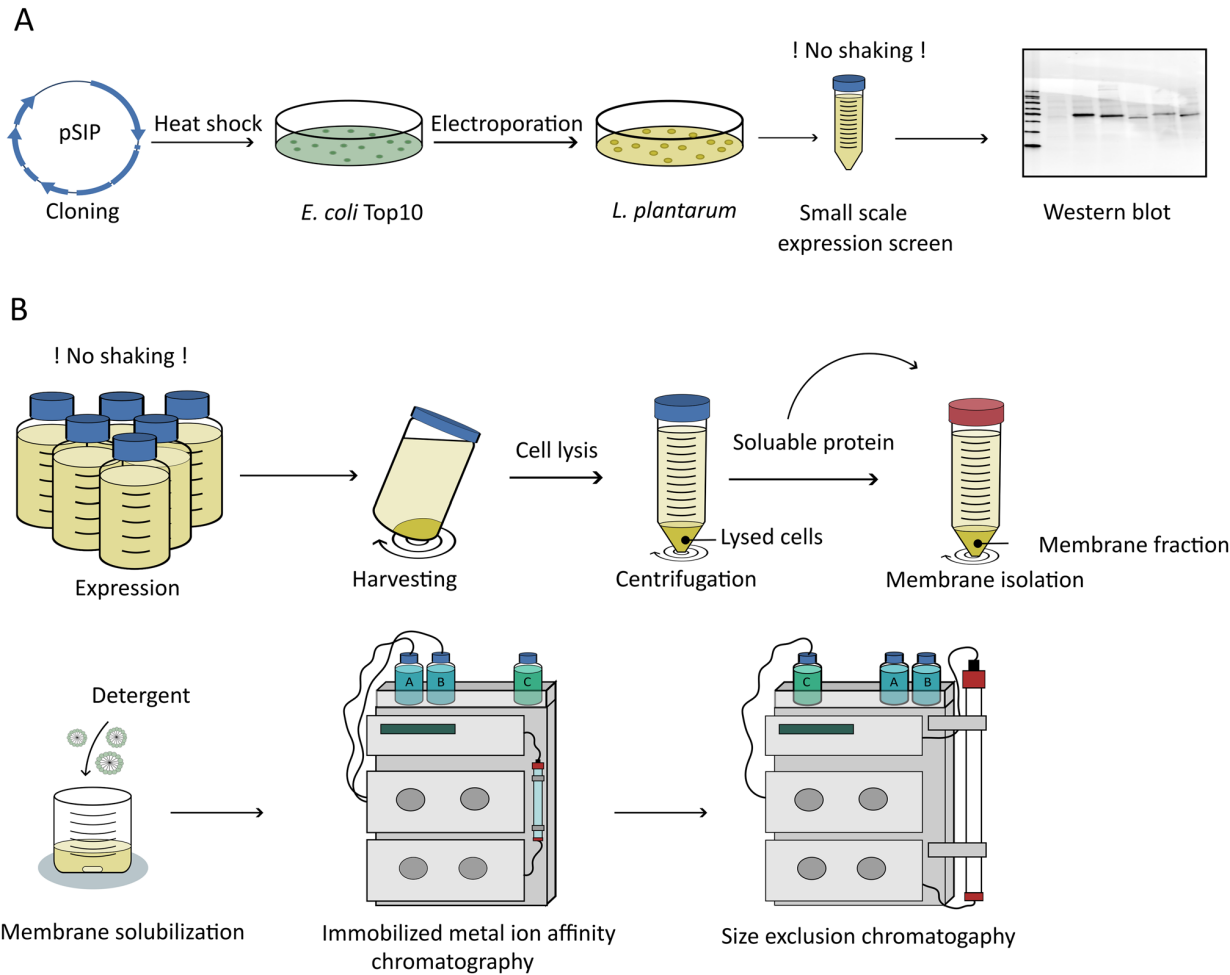
A schematic overview of the established workflow employed for construction, expression, and purification of recombinant RseP using *L. plantarum* as the expression host. (**A**) Construction of the plasmid and small-scale expression screening. (**B**) Scale-up of protein expression and two-step purification of recombinant RseP using immobilized metal affinity chromatography and size exclusion chromatography.

**Figure 4:**
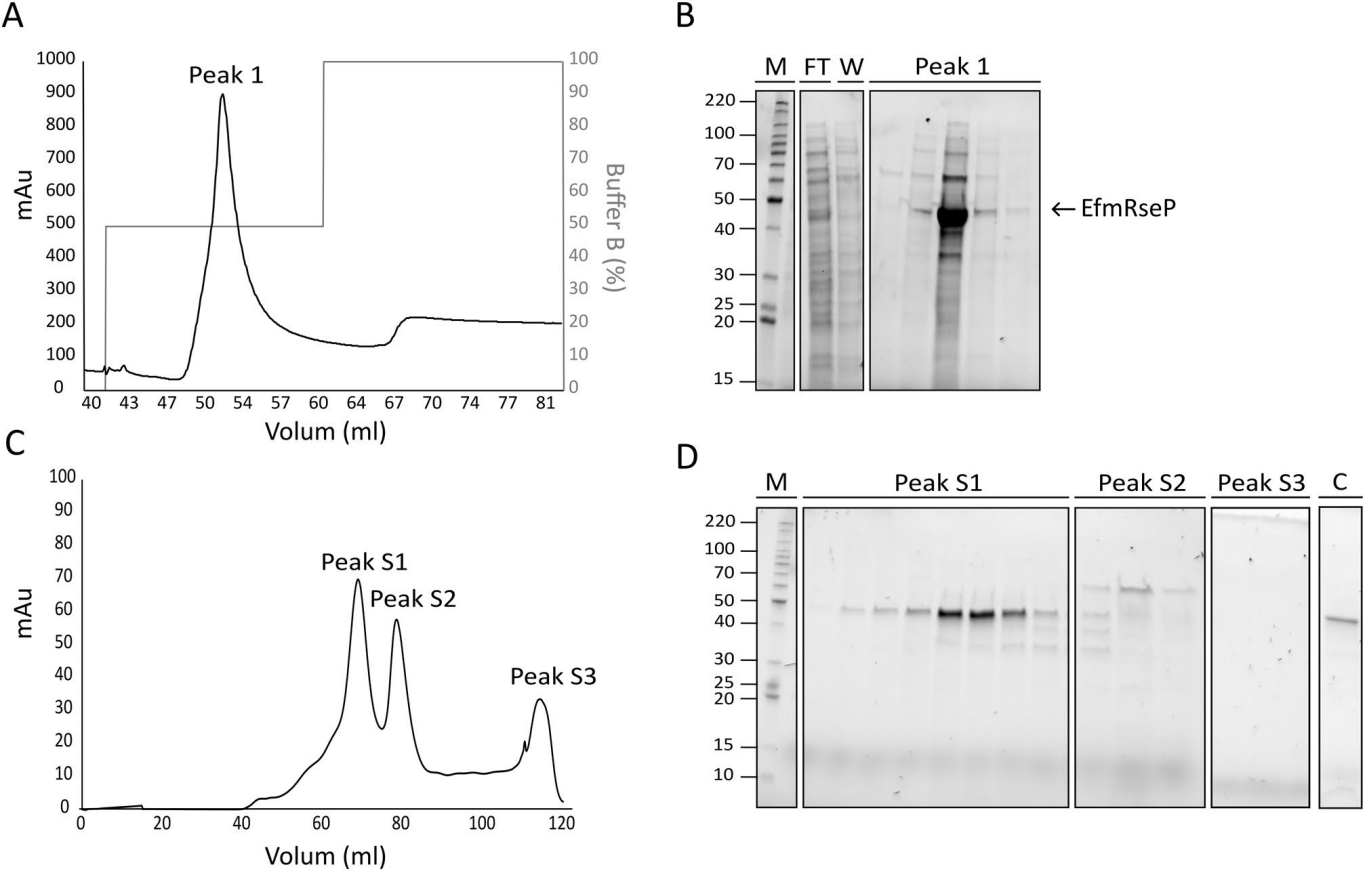
Overview of the two-step purification of EfmRseP using *L. plantarum* as the expression host. (**A**) A representative IMAC chromatogram profile showing the elution of EfmRseP from the NiNTA resin. (**B**) A 10 µl sample of the fractions from the flowthrough (FT), washing steps (W) and fractions corresponding to Peak 1 was analyzed using SDS-PAGE. Fractions containing EfmRseP (peak 1) were subsequently pooled and concentrated prior to being applied for SEC. (**C**) A representative SEC chromatogram profile indicated three size-exclusion peaks (S1, S2 and S3). (**D**) A 10 µl sample from the fractions from each peak was analyzed using SDS-PAGE. To analyze the purity of EfmRseP, a 1:10 dilution of the concentrated peak S1 was analyzed using SDS-PAGE (**C**). In both gels, EfmRseP runs at the expected size of 46 kDa. M indicates molecular mass marker in kDa. The full-length, original SDS–PAGE gels are presented in supplementary Fig. S7.

The optimal conditions identified in the small-scale (50 ml) production screen were applied for large-scale (1 L) production and purification of EfmRseP (25 ng/ml SppIP, 30 °C, 10 h). A schematic overview of the workflow is summarized in Fig. 3. EfmRseP was produced in 1 L tightly sealed Shott-Duran flasks, using a total of 6 L bacterial culture per round of purification. Following solubilization with n-Dodecyl β-maltoside (β-DDM), EfmRseP was purified by immobilized metal ion affinity chromatography (IMAC) (Fig. 3B). As seen in Fig. 4, EfmRseP bound strongly to the NiNTA-resin, while most contaminants were found in the flow-through and the wash fractions. EfmRseP was efficiently eluted using 50% Buffer B, resulting in considerable protein levels at the expected size (46 kDa) in peak 1 (Fig. 4A,B). However, several other bands were also visible in the same fraction, indicating contamination by other proteins. The EfmRseP containing fractions (peak1; Fig. 4B) were pooled, concentrated, and applied to size-exclusion chromatography (SEC) to ensure the purity and homogeneity of the sample. SEC resulted in three distinct peaks at 68.64 ml (peak S1), 78.46 ml (peak S2), and 114.50 ml (peak S3), respectively (Fig. 4C). SDS-PAGE analysis of the fractions in peak S1 (Fig. 4D) indicated considerable levels of EfmRseP, while the protein contaminants observed following IMAC purification was eluted in peak S2. No bands were observed in peak S3. Fractions corresponding to Peak S1 were collected and concentrated, resulting in a final yield of 0.35 mg EfmRseP per gram wet cell pellet. The identity of the purified protein was confirmed as *E. faecium* RseP using liquid chromatography tandem mass spectrometry (LC–MSMS; Fig. S3).

Comparison of the SEC elution profile with the elution profile of known protein standards allowed an estimation of the oligomeric state of EfmRseP. EfmRseP has an expected size of 46 kDa, however, the protein eluted at 68.64 ml, which is comparable to the retention volume of aldolase (67.36 ml, 158 kDa) (Fig. 4C,D, Fig. S4). β-DDM forms relatively large micelles (MW ap. 65–70 kDa)^48,49^, indicating that EfmRseP may exist as a dimer in the presented β-DDM concentration. It would be possible to get a more precise molecular size indication using SEC combined with Multi Angle Light Scattering (SEC_MALS). However since oligomeric state of many membrane proteins are dependent on the detergent to protein ratio and the detergent type, as exemplified for the Na^+^, K^+^-ATPase^50^, such a study would have to be combined with activity assays for the individual fractions to determine whether a dimer or higher oligomeric state is needed for activity. We believe this is beyond the scope of this paper.

### Large-scale purification of EfmRseP from *E. coli*

**Figure 5:**
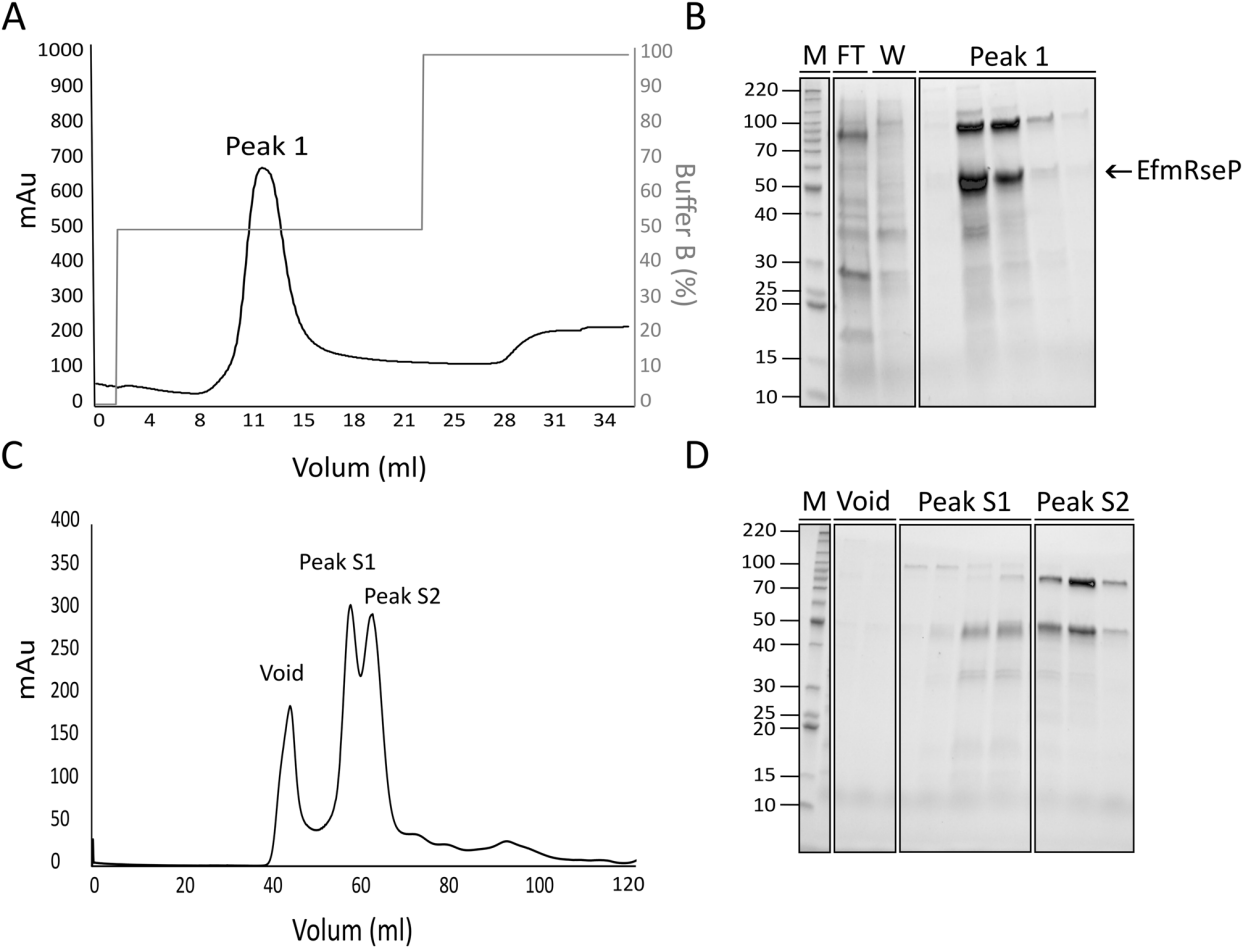
Overview of the two-step purification of EfmRseP expressed in *E. coli*. (**A**) A representative IMAC chromatogram profile showing the elution of EfmRseP from the NiNTA resin. (**B**) A 10 ml sample of fractions from the flowthrough (FT), the washing steps (W) and peak 1 was analyzed using SDS-PAGE. Appreciable amounts of EfmRseP were found in fractions corresponding to Peak 1. Peak 1 was pooled and concentrated prior to SEC analysis. (**C**) A representative SEC chromatogram. (**D**) Fractions from the void, peak S1 and peak S2 were analyzed using SDS-PAGE. The arrow indicates the expected size of EfmRseP (46 kDa). M indicates molecular mass marker in kDa. The full-length, original SDS-PAGE gels are presented in supplementary Fig. S8.

To evaluate *L. plantarum* as an expression and purification platform, EfmRseP expression and purification were attempted in the more traditional expression host *E. coli*. Using the standard IPTG inducible pET22b-vector, the detection of soluble EfmRseP failed in two out of three tested *E. coli* strains (Fig. S5). *E. coli* C43(DE3), a modified expression strain specifically developed for the expression of toxic proteins, was the only strain that gave a detectable amount of soluble EfmRseP. The highest protein production levels were observed after incubation overnight at 23 °C (Fig. S5). Purification of EfmRseP expressed in *E. coli* C43(DE3) was attempted using the same purification scheme as established for the purification of EfmRseP from *L. plantarum*. Figure 5A,B shows considerable levels of EfmRseP in Peak1 following IMAC-purification from production in *E. coli* C43(DE3). However, similar to purification from *L. plantarum,* several contaminating proteins were observed in the first purification step. SEC analysis of the Peak 1 fractions from the IMAC resulted in two overlapping peaks at 59.01 and 63.92 ml, respectively (Fig. 5C). One band at the expected size of EfmRseP was observed in both peaks (46 kDa), together with an additional band between 80 and 90 kDa and a weaker band of ap. 100 kDa (Fig. 5D). Using LC–MSMS analysis, the strong bands in peak 1 and peak 2 were identified as *E. faecium* RseP (Band 1, 46 kDa) and *E. coli* maltodextrin phosphorlylase (Band 2, 80–90 kDa; Fig. S3).

### Secondary structure analysis of purified EfmRseP

**Figure 6:**
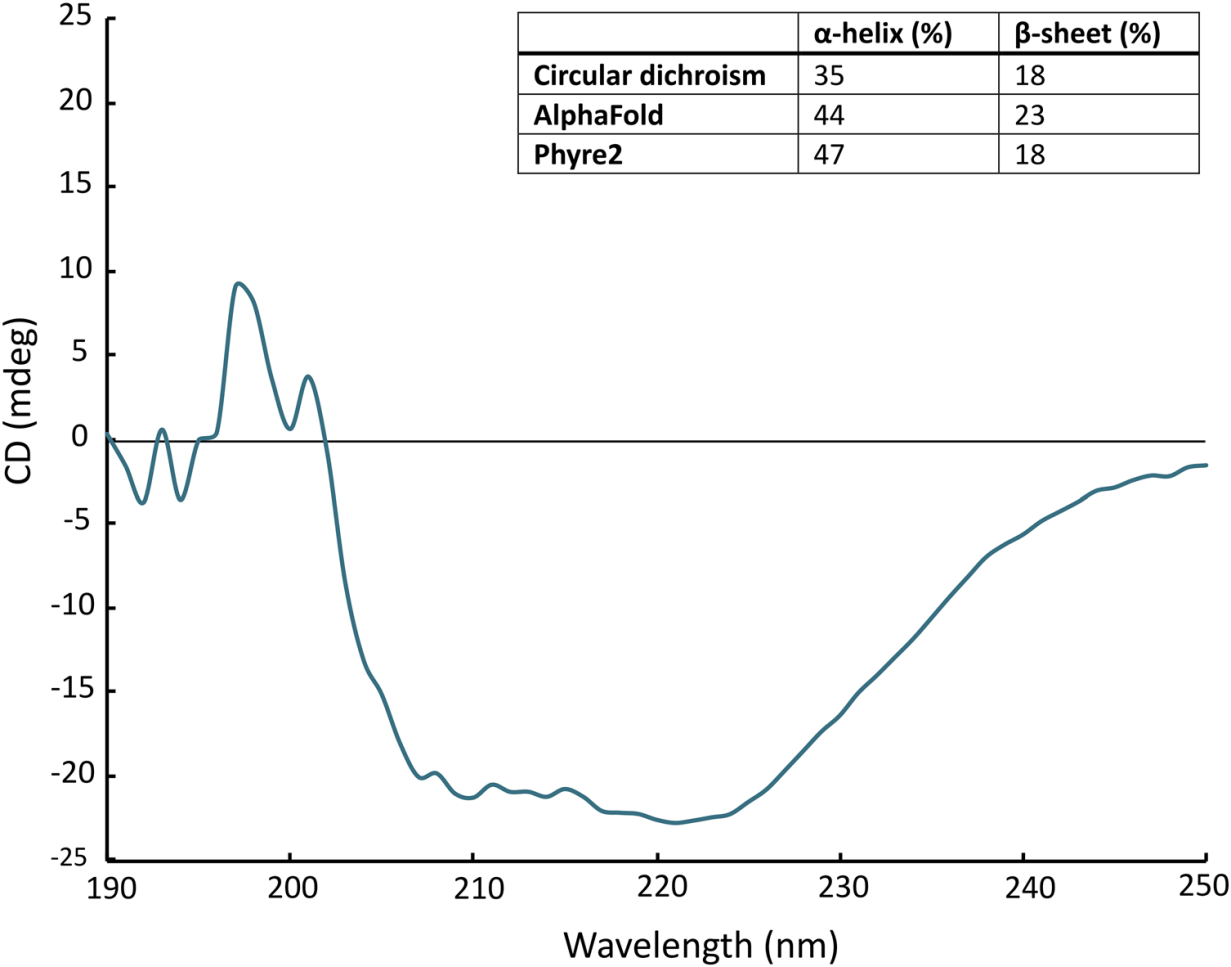
Secondary structure analysis using circular dichroism (CD) spectroscopy. CD spectra of the purified EfmRseP expressed in *L. plantarum*. The table (inset) shows the percentage of secondary structure of purified EfmRseP as estimated by Dichroweb and predicted by the structure prediction tools AlphaFold and Phyre2.

To investigate the protein folding following the purification of EfmRseP expressed in *L. plantarum*, the secondary structure was determined using circular dichroism (CD) analysis. The CD spectrum was analyzed using the online tool DicroWeb^51,52^, which indicated that the secondary structure of the protein was a mixture of α-helices and β-sheets (Fig. 6). Purified EfmRseP expressed in *L. plantarum* was estimated to contain 35% α-helix and 18% β-sheets. This is comparable to the secondary structure data predicted by Phyre2 and AlphaFold2 (Fig. 6), suggesting that the purified protein is folded correctly and suitable for further analysis. Due to failure to separate EfmRseP from contaminating protein (e.g. maltodextrin phosphorylase), the CD analysis of the EfmRseP expressed in *E. coli* C43(DE3) would have less value.

## Discussion

Membrane proteins remain one of the most challenging targets to characterize, with the overproduction of soluble proteins being the major hurdle in this pipeline. *E. coli* is the most extensively used host for the overproduction of recombinant membrane proteins (Fig. 1), however, poor stability, toxicity, and formation of inclusion bodies of the target protein are common problems^5,6^. In recognition of these limitations, several tags (e.g., Mistic, MBP) and optimized expression strains (e.g., C41(DE3), C43(DE3), Lemo21) are used to improve protein expression and solubility^6^. The membrane environment significantly impacts the stability of membrane proteins, while native cellular conditions and/or the presence of native chaperons may facilitate protein folding. Thus, screening different expression hosts can be an important strategy to achieve optimal production. In line with this notion, we evaluated both the Gram-positive *L. plantarum* and the more traditional expression host *E. coli* for expression and subsequent purification of the integral membrane protein RseP.

Five orthologs of RseP were successfully expressed in *L. plantarum,* with the RseP derived from *E. faecium* achieving the highest production levels at the conditions tested (Fig. 2A). The small-scale optimization using *L. plantarum* harboring pEfmRseP indicated that a 10-h incubation at 30 °C post-induction was optimal (Fig. 2B). EfmRseP was successfully purified using these conditions, yielding a total of 0.35 mg per gram wet cell pellet of highly pure protein (Fig. 4). This is comparable to yields achieved for membrane proteins produced and purified in *E. coli*^53^.

Previous studies have indicated 2–6 h post-induction as the optimal harvesting time for intracellular proteins using the pSIP system^26^. However, it has not been evaluated at expression conditions below 30 °C. We demonstrated that protein production time is temperature dependent in *L. plantarum*, as induction is prolonged and protein degradation is reduced at lower temperatures (23 °C and 30 °C). It should be noted that although harvesting cells incubated at 30 °C 10 h post-induction was suggested as optimal, the protein was also successfully obtained following incubation at room temperature overnight (0.24 mg pellet per gram wet cell pellet). The facultative anaerobic nature of *L. plantarum*, and its ability to produce a large amount of proteins at room temperature, suggests that this method could be suitable for industrial-scale production in bioreactors.

We also assessed the production of EfmRseP in three well-known production strains of *E. coli* (Fig. S5). While all three strains are especially modified for overexpression of proteins, the detection of soluble protein expression failed in two of the three strains tested. *E. coli* C43(DE3) produced considerable levels of EfmRseP; however, the same two-step purification as applied for EfmRseP produced in *L. plantarum* did not result in a pure, monodispersed protein (Fig. 5). Two strong protein bands were observed in peak S2 following SEC-analysis: one band at ap. 45 kDa (expected size of EfmRseP), and one band between 80 and 90 kDa. Using LC-MSMS the band of the lower molecular weight was identified as RseP from *E. faecium,* while the band between 80 and 90 kDa was identified as *E. coli* maltodextrin phosphorylase (Fig. S3). *E. coli* have previously been successfully used to express and purify RseP orthologs from the thermophile archaea *Methanocaldococcus jannaschii* (MjS2P), the marine bacterium *Kangiella koreensis* (KkRseP) and *E. coli* (EcRseP)^45,47^. However, it should be notated that only the core region of MjS2P has been purified, as the full-length protein was found to be highly susceptible to degradation following purification^45^. While the full-length KkRseP and EcRseP has been purified, the purification protocol is elaborate, requiring multiple rounds of SEC-purification to obtain a pure and monodispersed sample^47^.

To evaluate the folding of a recombinant protein, the biological activity of the purified protein is often tested. Previous studies have exploited the known substrates of RseP orthologs to perform a cleavage-based assay to confirm proper protein expression and folding^47,54,55^. However, the RseP substrate in *E. faecium* is unknown. Moreover, lipids are known to influence the activity of purified membrane proteins, making activity assays challenging to establish for membrane proteases^56–58^. To circumvent these difficulties, we investigated protein folding and functional expression of RseP using two different approaches. First, a spot-on-lawn assay was conducted on whole *L. plantarum* cells to confirm the proper expression and folding of all RseP orthologs in vivo (Fig. S1)*.* A key advantage of Gram-positive expression hosts is that they are monoderm, allowing direct evaluation of membrane protein targeting inhibitors or substrates, without the need to circumvent the outer membrane of Gram-negative hosts. All RseP orthologs, except for LpRseP, acts as a receptor for the LsbB family of bacteriocins in their native organisms. When screen against EntK1 in the spot-on-lawn assay, *L. plantarum* cells expressing RseP orthologs from EntK1-senstivite species (e.g. EfmRseP, EfsRseP, ShRseP and LlRseP) conveyed EntK1-senstivity to the EntK1-resistant host *L. plantarum,* suggesting the proteins where functionally expressed in vivo (Fig. S1). Members of the LsbB bacteriocin family has only been shown to inhibit Gram-positive bacteria^40,41^. The lack of activity against Gram-negative cells is suggested to be attributed to limited access to RseP due to the outer membrane, thus the spot-on-lawn assay cannot be applied to confirm proper expression and folding when expressing RseP in *E. coli* or other Gram-negative host.

Secondly, we investigated the secondary structure of the purified EfmRseP using CD analysis, a commonly used technique for the determination of secondary structure and folding properties of recombinantly expressed proteins. Using the Dichroweb analysis tool, purified EfmRseP expressed in *L. plantarum* was estimated to contain 35% α-helices and 18% β-sheets which is comparable to the estimated secondary structure predicted by Phyre2 and AlphaFold2, indicating that the protein is properly folded (Fig. 6).

In summary, we present the pSIP expression system and *L. plantarum* as a novel platform for the production of pure and soluble membrane proteins (Fig. 3). In the specific case of EfmRseP, expression and purification of EfmRseP from *L. plantarum* was superior, requiring fewer purification steps to reach a pure, monomeric protein compared to expression and purification in *E. coli*. It should be notated that *E. coli* strains such as *E. coli* C43(DE3) is highly tailored for protein expression, while *L. plantarum* is not specifically modified for overexpression of proteins. Still, lactic acid bacteria offer some clear advantages as expression host compared to *E. coli*, including low proteolytic pressure, small genome size and a monoderm cell envelope. The results presented in this work highlights *L. plantarum* and the pSIP system as an alternative and promising expression platform, thereby expanding the current toolbox available for protein overexpression.

## Materials and methods

### Bacterial strains and cultivation conditions

The bacterial strains used in this study are listed in Table 1. Enterococcal strains and *Staphylococcus haemolyticus* were cultivated in brain heart infusion (BHI) broth (Thermo Scientific™, Oxoid™) at 37 °C. All *E. coli* strains were cultivated in Tryptic Soy Broth (TBS) or BHI broth (Thermo Scientific™, Oxoid™) at temperatures ranging from 12 to 37 °C, depending on the experiment. *Lactococcus lactis* was cultivated in M17 broth (Thermo Scientific™ Oxoid ™) supplemented with 0.5% glucose at 30 °C while *L. plantarum* was cultivated in De Man, Rogosa and Sharpe (MRS) broth (Thermo Scientific™, Oxoid™) at temperatures ranging from 23 to 37 °C, depending on the experiment. The enterococcal strains, *L. lactis* and *L. plantarum* were cultivated without shaking, while *E. coli* strains and *S. haemolyticus* were cultivated with agitation at 190 rpm.

**Table 1:** Plasmids and bacterial strains used in this study.

Strain or plasmid	Relevant characteristic(s)	References
Plasmids		
pLp1261_InvS	pSIP-based expression vector, pSIP401 backbone, Ery^R^	^27,59^
pET22b_EfmRseP6His	pET-based expression vector, TEV cleavable C-terminal 6xHis-tag, Amp^R^	This study/Genscript
pEV	Empty vector, containing the regulatory genes and promoter of the pSIP system, but no gene downstream of the inducible promoter	^59^
pEfmRseP	pSIP401 derivative containing *rseP* derived from *E. faecium* P21. C-terminal 6xHis-tag	^43^
pLpRseP	pSIP401 derivative containing native *L. plantarum* WCSF1 *rseP*. C-terminal 6xHis-tag	^43^
pEfsRseP	pSIP401 derivative containing *rseP* derived from *E. faecalis* V583. C-terminal 6xHis-tag	This study
pLlRseP	pSIP401 derivative containing *rseP* derived from *L. lactis* IL1430. C-terminal 6xHis-tag	This study
pShRseP	pSIP401 derivative containing *rseP* derived from *S. haemolyticus* 7076_4_21. C-terminal 6xHis-tag	This study
Strains		
* E. faecalis* V583 (*Efs*)	Template for *rseP* (*EfsrseP*)	NCBI:txid226185
* L. lactis* IL1403 (*Ll*)	Template for *rseP* (*LlrseP*)	NCBI:txid272623
* S. haemolyticus* 7076_4_21 (*Sh*)	Template for *rseP* (*ShrseP*)	ENA: ERS066353
*L. plantarum* WCFS1	Expression host	^63^
*E. coli* TOP10	Cloning host	ThermoFisher Scientific
*E. coli* artic express	Expression host, suitable for low growth temperatures	Agilent Technologies
*E. coli* BL21 Star™ (DE3)	Expression host	ThermoFisher Scientific
*E. coli* C43 (DE3)	Expression host, suitable for expression of toxic proteins	Sigma-Aldrich

Agar plates were prepared by supplementing the appropriate broth with 1.5% (w/v) agar (VWR chemicals). When appropriate, gentamycin, ampicillin or erythromycin were added to a final concentration of 20 µg/ml, 200 µg/ml, and 200 µg/ml, respectively, for *E. coli*, while erythromycin was added to a final concentration of 10 μg/mL for *L. plantarum.*

### Plasmid construction

Five orthologs of the *rseP* gene were expressed in *L. plantarum* using the pSIP expression system^27,28^ (Table 1). The construction of pEfmRseP and pLpRseP containing *rseP* derived from *Enterococcus faecium* P21 and *L. plantarum* WCFS1, respectively, has previously been described^43^. The *rseP* gene from *L. lactis* IL1430 (*LlRseP*), *S. haemolyticus* 7076_4_21 (*ShRseP*), and *Enterococcus faecalis* V583 (*EfsRseP*), was amplified using genomic DNA as a template for PCR amplification with target specific In-Fusion primers (Table S1) that yielded amplicons with complementary ends to a linearized vector. A 6xHis-tag was included in all reverse primers, thereby incorporating a C-terminal 6xHis-tag in the final protein product. The resulting PCR fragments were inserted into *Nde*I and *Acc*65I (both ThermoFisher Scientific™) digested pLp_1261_InvS^59^, a pSIP401 derivative, following the In-Fusion protocol (Takara Bio Inc, Goteborg, Sweden). The In-Fusion mix was transformed to competent *E. coli* TOP10 (ThermoFisher Scientific™) as described by the manufacturers. The resulting three plasmids (pLIRseP, pShRseP and pEfsRseP) were isolated from *E. coli* using the NuceloSpin Plasmid Kit (Macherey-Nagle) and verified by sequencing at Eurofins GATC Biotech (Germany) and subsequently transformed to electrocompetent *L. plantarum* WCSF1 as previously described^60^. An overview of the constructed plasmids is given in Table 1.

### Small-scale expression screen

A small-scale expression screen was conducted to determine optimal protein expression conditions using *L. plantarum*. Briefly, an overnight culture was diluted in falcon tubes containing 50 ml pre-warmed MRS to an OD_600_ of 0.10–0.15 followed by incubation at 37 °C without agitation. When reaching an OD_600_ of 0.27–0.35, the cultures were induced with concentrations of SppIP ranging from 12.5 to 50 ng/ml and incubated for 3-, 6-, 10- or 24 h before harvesting. The small-scale expression screen was performed at three temperatures: 23 °C, 30 °C and 37 °C. The cultures were harvested by centrifugation for 10 min at 5000×*g* (4 °C). Harvested cell pellets were stored at − 20 °C prior to western blot analysis. A schematic overview of plasmid construction and expression optimization is given in Fig. 3A.

### Plasmid construction and expression in *E. coli*

A codon-optimized *EfmRseP* was synthesized and inserted into the pET22b vector by GenScript, resulting in the plasmid pET22b-EfmRseP6His. The plasmid was transformed into the three *E. coli* strains (Table 1) according to the manufacturer’s instruction. All clones were verified with colony PCR using the Red Taq DNA polymerase Master Mix (VWR).

For the overproduction of RseP in *E. coli,* two different growth media (BHI and TBS) were tested, and the bacteria were harvested at two different time points (3- and 24 h after induction). The expression protocol was based on the protocol provided by the manufacturer, as well as protocols from previous expression studies of RseP derived from Gram-negative bacteria in *E. coli*^45^. Briefly, an overnight culture was diluted to an OD_600_ of ~ 0.1. *E. coli* BL21 Star™ (DE3) Star and *E. coli* C43(DE3) were incubated at 37 °C, while *E. coli* ArcticExpress was incubated at 30 °C. All strains were incubated with agitation until reaching an OD_600_ of 1.0–1.5. *E. coli* ArcticExpress was incubated with agitation at 10 °C for 10 min before induction with IPTG to a final concentration of 1 mM, while *E. coli* BL21 Star™ (DE3) and *E. coli* C43(DE3) were induced directly with IPTG to a final concentration of 200 µM. *E. coli* ArcticExpress was incubated at 10 °C post-induction, while *E. coli* BL21 Star™ (DE3) and *E. coli* C43(DE3) were incubated at 23 °C post-induction. The cells were harvested by centrifugation (5000×*g*, 10 min, 4 °C), at 3- and 24 h post-induction and stored at − 20 °C before western blot analysis.

### Western blot analysis

Western blot analysis was performed to investigate protein production levels at the various expression conditions. *E. coli* and *L. plantarum* harboring expression plasmids were lysed and applied to a SDS–PAGE as previously described^31^, with minor adjustments. Briefly, harvested cells were resuspended in 250 µl NP-40 buffer (150 mM NaCl; 1.0% Triton X-100; 50 mM Tris, pH 8.0) containing 1 mM phenylmethylsulfonyl fluoride (PMFS) and lysed using a FastPrep FP120 Cell Disrupter (MP Biomedicals, Santa Ana, CA). *L. plantarum* cells were lysed using three cycles of shaking at 6.5 m/s for 30 s, while *E. coli* cells were lysed using a single cycle of shaking at 6.0 m/s for 30 s. The volume of the protein extract applied to the SDS–PAGE was normalized based on OD_600_ at the harvesting time point, if not otherwise stated. Following electrophoresis, the proteins were blotted to a nitrocellulose mini membrane using iBlot™ Transfer Stack (Invitrogen) and the iBLot™ Gel transfer device (Invitrogen). The membrane was washed with Tris-buffered Saline (TBS) (2 × 10 min), prior to blocking (1 h, 5% Bovine Serum Albumin (BSA)). Following blocking, the membrane was washed (2 × 10 min Tween-TBS (TTBS; 0,05% Tween-20), 1 × 10 min TBS), prior to incubation with the primary antibody (Penta-His™ (Qiagen), 1:1000 in 5% BSA) for 30 min at room temperature. The membrane was subsequently incubated with the primary antibody at 4 °C overnight, followed by a 30 min incubation at room temperature the following day. The membrane was washed (2 × 10 min, TTBS), and incubated with the HRP-conjugated polyclonal anti-mouse IgG (Sigma-Aldrich) secondary antibody, diluted 1:5000 in the blocking buffer, for 1 h. Unbound secondary antibodies were removed by washing 4 × 10 min with TTBS. All incubations and washing steps were performed with agitation. The blots were visualized using the SuperSignal West Pico PLUS Chemiluminescent substrate (Thermo Fisher Scientific) and the Azure c400 system (Azure Biosystem, Dublin, CA). Band intensity was estimated using ImageJ and protein concentration was estimated based on the known concentration of the positive control^61^.

### Antimicrobial assays

To verify RseP production in *L. plantarum*, the antimicrobial activity of bacteriocins targeting RseP was assessed using a spot-on-lawn assay. Briefly, an overnight culture was diluted in soft-agar (1:1000) and distributed on agar plates containing erythromycin. Once solidified, bacteriocins were applied to designated spots. All bacteriocins used in this study were produced by Pepmic Co., Ltd (Suzhou, China) with > 95% purity, and solubilized in 0.1% (vol/vol) trifluoroacetic acid (TFA; Sigma Aldrich). Agar plates were incubated at appropriate temperatures overnight and inhibition zones without bacterial growth were measured the following day.

### Purification of recombinant EfmRseP

The conditions used during large-scale production of EfmRseP were based on the results from the small-scale optimization. Due to the facultative anaerobic nature of *L. plantarum*, the bacteria were cultivated in 1 L Schott Duran bottles (30 °C, 10 h). *E. coli* was cultivated in Erlenmeyer flasks to ensure proper aeration (23 °C, overnight). The cells were harvested by centrifugation (1227×*g*, 4 °C, 20 min), and stored at − 20 °C.

The protein was purified as previously described^53^, with minor modifications. Briefly, cell pellets were resuspended in a 1:10 ratio in lysis buffer (50 mM HEPES, pH 7.0; 200 mM KCl, 10% Glycerol, 1 mM PMFS, 1 unit DNase I (ThermoFisher Scientific)/10 ml buffer. *L. plantarum* cells were lysed using an LM20 Microfluidizer® Processer (Microfluidics®) using two passages at 30,000 PSI, while *E. coli* cells were lysed using a single passage at 15 000 PSI. Intact cells were pelleted by centrifugation (8000×*g*, 4 °C for 20 min). The supernatant was subsequently centrifuged at 150,000×*g* for 1 h at 4 °C using the Sorvall™ WX ultracentrifuge (ThermoFisher Scientific™). The resulting pelleted membrane was resuspended in a 1:20 ratio in membrane resuspension buffer (50 mM HEPES, pH 7.0; 200 mM KCl, 10% Glycerol, 1 mM PMFS) using a Potter–Elvehjem PTFE pestle (Sigma-Aldrich). The solubilized membrane was aliquoted and flash-frozen in liquid nitrogen or directly solubilized in 1% β-DDM (ap. 1 h, 200 rpm, 4 °C). A 5 ml HisTrap™ HP column (Cytiva) was equilibrated with 4 × column volume (CV) of buffer A (50 mM Hepes, pH 7.0; 200 mM KCl; Imidazole 20 mM; Glycerol 10%, 3 × CMC β-DDM, 1 PMFS) before the solubilized membrane suspension was loaded onto the column at 0.5 ml/ml flow rate using the Äkta Start protein purification system.

The column was washed with buffer A until the UV was reduced to 50 mAU at a 1 ml/min flow rate. The protein was eluted with 4 CV of 50% buffer B (50 mM HEPES, pH 7.0; 200 mM KCl; Imidazole 500 mM; Glycerol 10%, 3 × CMC β-DDM, 1 PMFS) with a flow rate at 2 ml/min. The eluted fractions containing the target protein were pooled and concentrated to ≤ 1 ml using the Amicon® Ultra Centrifugal filters (Sigma-Aldrich), before being subjected to size-exclusion chromatography using a HiLoad® 16/600 Superdex® 200 pg (Sigma-Aldrich) with buffer C (25 mM HEPES, pH 7.0; 200 mM KCl; Glycerol 5%, 3 × CMC β-DDM,). Standards from the high molecular weight Gel filtration calibration kit from Cytiva (Marlborough, MA, USA) was used to estimate the protein size. Fractions from size exclusion were pooled, concentrated, flash-frozen in liquid nitrogen and stored at − 80 °C until use. A schematic overview of the purification process is given in Fig. 3B.

### Circular dichroism spectroscopy

All circular dichroism spectroscopy (CD) experiments were performed on a Jasco CD J1500 circular dichroism spectropolarimeter, equipped with a Peltier-controlled cuvette holder. The CD analysis was performed to determine the secondary structure of the purified protein. The purified protein was diluted in buffer (12.5 mM HEPES, pH 7.0; 200 mM KCl; 2.5% glycerol) to a final concentration of 0.2–0.24 mg/ml. Spectral data were obtained at 20 °C, at 100 nm/min scan rate and 1 nm bandwidth. A cuvette with a 0.1 cm path length was applied. The resulting spectra represent five scans from 190 to 250 nm. Dichroweb was used to estimate secondary structure using the K2D method^51,52^. The protein sample used in CD-analysis were harvested following overnight production at room temperature. EfmRseP was purified as described above, except for using a ProteoSEC Dynamic 16/60 3–70 HR column (ProteinArk) for SEC-analysis.

### Liquid chromotagraphy tandem mass spectrometry

EfmRseP purified from *L. plantarum* (Peak S1) and from *E. coli* C43(DE3) (Peak S1 and S2) was applied to an SDS-PAGE. The band of interest was cut and destained using 50 mM Amic solution with 50% Acetonitrile (ACN). Using 100% ACN, the gel pieces were dehydrated (10 min, shaking). The ACN was subsequently removed, and the gel pieces were incubated in reduction solution (10 mM tris(2-carboxyethyl)phosphine hydrochloride (TCEP), 40 mM chloroacetamide (CAA), 80 mM Ambic) for 30 min with agitation. Following reduction and alkylation, the sample is dehydrated as described above. The samples are incubated on ice for 10 min with trypsin solution (25 ng/μL Trypsin in 50 mM Ambic, 10% ACN). 100 mM Ambic is subsequently added to cover the gel pieces and the samples are incubated at 37 °C over night. The following day, peptides are extracted by adding 50 μL extraction buffer (5% formic acid (FA), 50% ACN) and incubated at 37 °C for 30 min. The supernatant is removed and the extraction step is repeated. The supernatant from the extractions are combined and dried before resuspension in loading solution (2% ACN, 1% TFA).

For each sample, peptides were loaded onto a 2 cm C18 trap column (ThermoFisher 164946), connected in-line to a 15 cm C18 reverse-phase analytical column (Thermo EasySpray ES904) using 100% Buffer A (0.1% FA in water) at 750 bar, using the Thermo EasyLC 1200 HPLC system, and the column oven operating at 45 °C. Peptides were eluted over a 45 min gradient ranging from 10 to 60% of 80% ACN, 0.1% FA at 250 nl/min, and the Q-Exactive instrument (Thermo Fisher Scientific) was run in a DD-MS2 top10 method. Full MS spectra were collected at a resolution of 70,000, with an AGC target of 3 × 10^6^ or maximum injection time of 20 ms and a scan range of 300–1750 m/z. The MS2 spectra were obtained at a resolution of 17,500, with an AGC target value of 1 × 10^6^ or maximum injection time of 60 ms, a normalised collision energy of 25 and an intensity threshold of 1.7e^4^. Dynamic exclusion was set to 60 s, and ions with a charge state < 2 or unknown were excluded. MS performance was verified for consistency by running complex cell lysate quality control standards, and chromatography was monitored to check for reproducibility.

The raw files were analysed using Proteome Discoverer 2.4. The spectra were matched against the Uniprot database of *E. coli* (UP000000625), *L. plantarum* (UP000000432) and the RIP metalloprotease RseP protein sequence from *E. faecium*. Dynamic modifications were set as Oxidation (M) and Acetyl on protein N-termini. Cysteine carbamidomethyl was set as a static modification. All results were filtered to a 1% FDR.

### Statistics and bioinformatics

To evaluate the prevalence of Gram-positive bacteria as an expression host for IMPs, a custom tabular report was downloaded from the Protein data bank on 18.10.2022. All experimental entries annotated as transmembrane proteins (PDBTM) were selected and sorted based on the expression host. All unpublished entries and entries with no defined expression host were removed. NCBI taxonomy browser determines the taxonomy of the expression organism. To validate the secondary structure determined using CD analysis, the EfmRseP structure was predicted using the protein fold recognition server Phyre 2^62^. The previously published predicted AlphaFold2 structure of EfmRseP was used as an additional validation control^43^.

### Supplementary Information

Supplementary Information.

## Data Availability

All data is available upon request. Please contact corresponding author. The raw data and search output from the LC-MSMS analysis is uploaded to the ProteomeXchange partner MassIVE (https://massive.ucsd.edu/ProteoSAFe/static/massive.jsp) with the identifier MSV000092087.
