# Supplementary material for: Lactiplantibacillus plantarum as a novel platform for production and purification of integral membrane proteins using RseP as the benchmark
Source: Sci Rep. 2023 Sep 1;13:14361. doi: 10.1038/s41598-023-41559-7 (PMC10474122; doi:10.1038/s41598-023-41559-7)
Supplement: Supplementary file 1 — Supplementary Information. [file 41598_2023_41559_MOESM1_ESM.pdf]

# *Lactiplantibacillus plantarum* as a novel platform for production and purification of integral membrane proteins using RseP as the benchmark

Sofie S. Kristensen[1*], Marie V. Lukassen[2], Suzana Siebenhaar[2], Dzung B. Diep[1†], J. Preben Morth[2*] and Geir Mathiesen[1*].

[1] Faculty of Chemistry, Biotechnology and Food Science, Norwegian University of Life Sciences (NMBU), Ås, Norway

[2] Department of Biotechnology and Biomedicine, Technical University of Denmark (DTU), Kongens Lyngby, Denmark

* Address correspondence to: Sofie S. Kristensen (sofie.kristensen@nmbu.no) J. Preben Morth (premo@dtu.dk) or Geir Mathiesen (geir.mathiesen@nmbu.no).

† This author is deceased (7th of December 2022).

# Supplementary

**Table S1| Primers used in this study.**

| Primers | Sequence (5'-3')* | Ref. |
|---|---|---|
| EFs_V583_RseP_F | GGAGTATGATTCATATGAAAACAATTATCACATTCATTATT | This study |
| EFs_V583_Rsep_6HIS_R | TCGAACCCGGGGTACCTTAAT**GATGATGATGATGATGAT**GAAAGAAAAAGCGTTGAAT | This study |
| IL1403JRseP_F | GGAGTATGATTCATATGATAGAAACACTGATTACTTTTATT | This study |
| IL1403J_RseP6HIS_R | TCGAACCCGGGGTACCTTAAT**GATGATGATGATGATGAT**TTACAAAGGCTCGGAGAATATC | This study |
| SH_RseP_F | GGAGTATGATTCATATGAGCTATTTAATCACTATTGTCTCATTT | This study |
| SH_RseP6His_R | TCGAACCCGGGGTACCTTAAT**GATGATGATGATGATGAT**GCAAGAAATAACGTTGTATATCGTTC | This study |

* Introduction of the His-tag is marked in **bold**.

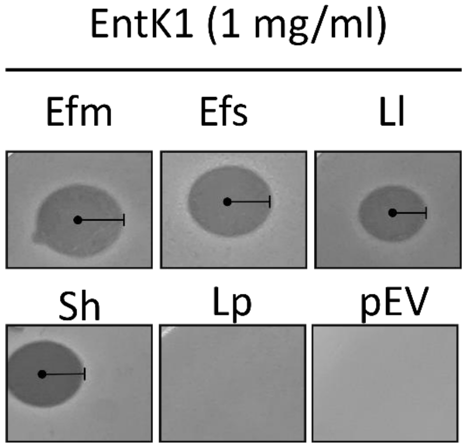


**Figure S1| Inhibition zone of EntK1 in a spot-on-lawn assay.** A 3 µl drop of EntK1 (1 mg/ml) was spotted on lawns of *L. plantarum* harboring different *rseP* orthologs. *L. plantarum* is naturally resistant towards EntK1, while *E. faecium* (Efm), *E. faecalis* (Efs), *L. lactis* (Ll) and some *S. haemolyticus* (Sh) strains are sensitive to EntK1. EntK1 depends on RseP as a receptor for its antimicrobial activity, thus increased sensitivity towards EntK1 compared to the empty vector (pEV) indicates expression of a functional receptor protein. All strains, except for the strain harboring the empty vector (pEV) and the ortholog derived from the host itself (Lp), exhibit an inhibition zone. The approximate diameter of the inhibition zone is indicated.

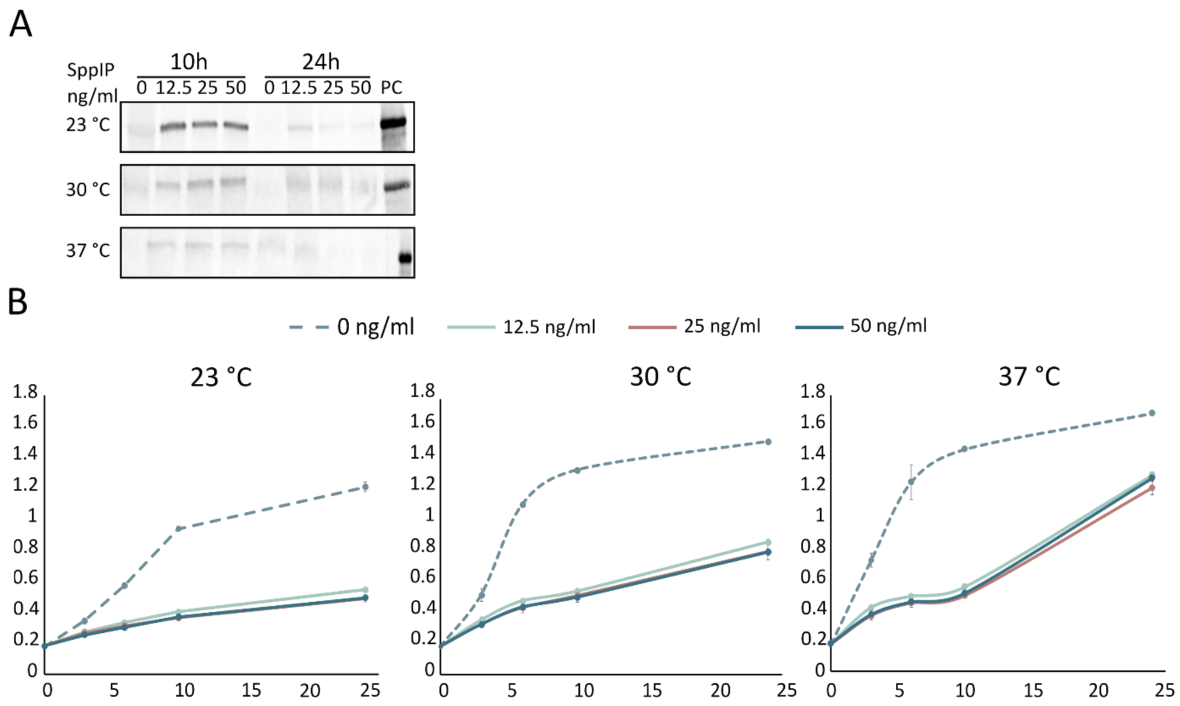


**Figure S2| Effect of inducer concentration on protein production and growth.** A) Western blot analysis showing the effect of inducer concentration on pro-longed protein expression (10- and 24 hours post-induction) at 23 °C, 30 °C and 37 °C. Purified EfmRseP was used as a positive control (PC). The full-length, original western blots are presented in supplementary Figure S9. B) Effect of increased inducer concentration on the growth rate at 23 °C, 30 °C and 37 °C. Non-induced sample is indicated by a dashed line. $OD_{595}$ was measured at induction (0 hour), as well as 3-, 6-, 10- and 24 hours post-induction. The experiment was performed with three biological replicates. Standard deviation is indicated for each data point.

52

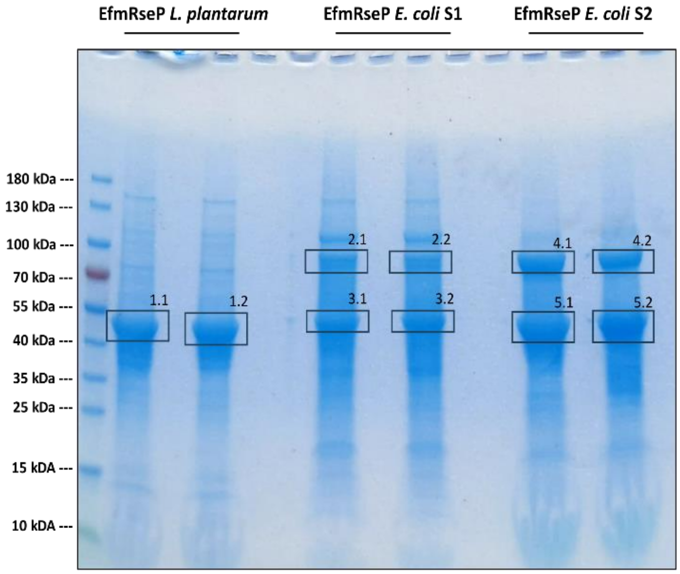


| Gel band | Protein | Organism | Coverage (%) | Peptides | PSM | Sequest HT Score |
|---|---|---|---|---|---|---|
| 1.1 | RIP metalloprotease RseP | *Enteroococcus facium* | 90 | 57 | 422 | 4559.27 |
| 1.2 | RIP metalloprotease RseP | *Enteroococcus facium* | 91 | 56 | 933 | 4071.78 |
| 2.1 | Maltodextrin phosphorylase | *Escherichia coli* | 93 | 98 | 824 | 3594.88 |
| 2.2 | Maltodextrin phosphorylase | *Escherichia coli* | 94 | 99 | 824 | 3490.93 |
| 3.1 | RIP metalloprotease RseP | *Enteroococcus facium* | 90 | 57 | 776 | 3478.83 |
| 3.2 | RIP metalloprotease RseP | *Enteroococcus facium* | 99 | 57 | 728 | 3267.11 |
| 4.1 | Maltodextrin phosphorylase | *Escherichia coli* | 92 | 98 | 864 | 3442.03 |
| 4.2 | Maltodextrin phosphorylase | *Escherichia coli* | 93 | 102 | 102 | 3405.82 |
| 5.1 | RIP metalloprotease RseP | *Enteroococcus facium* | 90 | 43 | 355 | 1291.39 |
| 5.2 | RIP metalloprotease RseP | *Enteroococcus facium* | 60 | 30 | 257 | 1050.49 |

**Figure S3| Identification of gel bands by LC-MSMS.** SDS-PAGE of the purification of EfmRseP from *L. plantarum* (Peak S1) and *E.coli* (Peak S1 and S2) The bands of interest at 46 kDa and 80-90 kDa were digested with trypsin and analysed using LC-MSMS. The top-ranking protein identification of each gel band is listed in the table with sequence coverage, number of peptides, number of peptide-matched-spectra (PSM), and Sequest HT score.

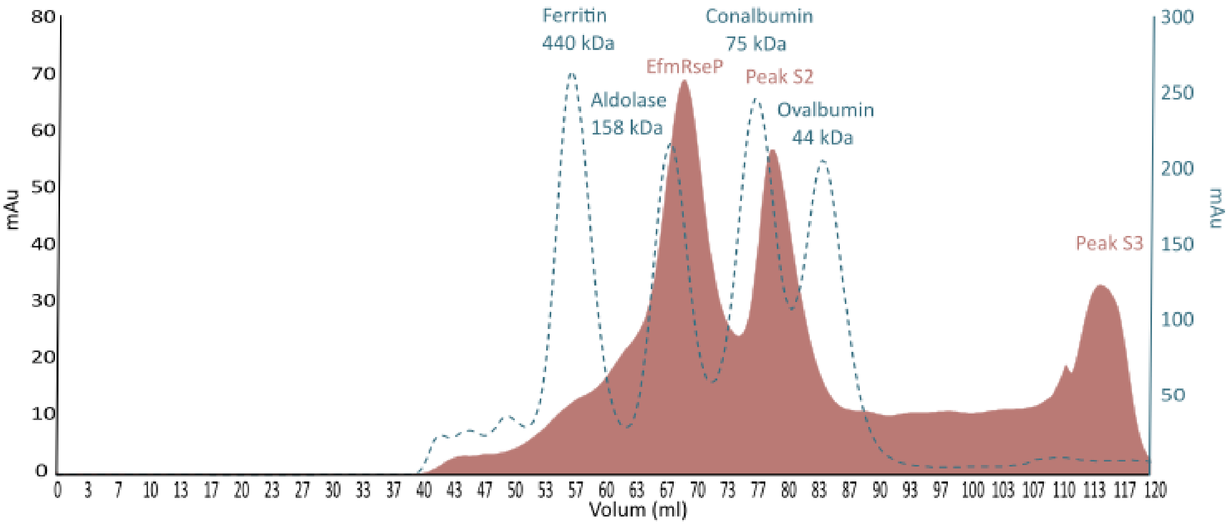


**Figure S4 |Chromatographic separation of standard proteins (dashed line) and EfmRseP on a HiLoad® 16/600 Superdex® 200 pg.**

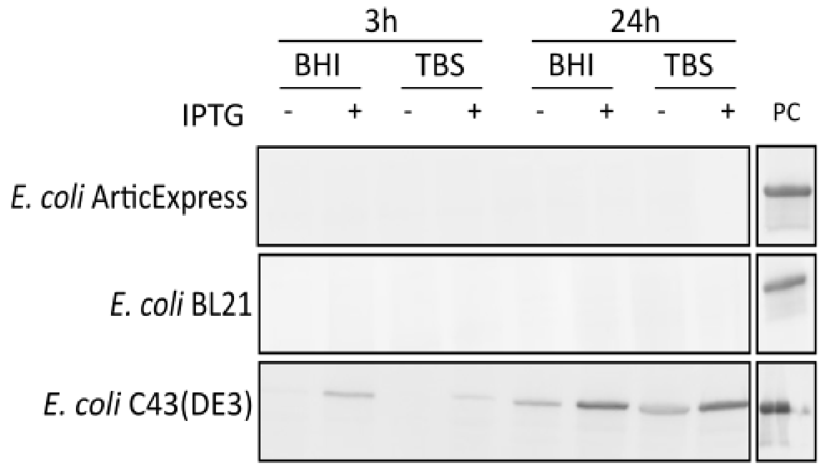


**Figure S5 | Western blot analysis of protein production in *E. coli* harboring pET22b-EfmRseP.** The small-scale expression screen was performed using three strains: *E. coli* Artic Express, *E. coli* BL21 Star™ (DE3), and *E. coli* C43(DE3). Expression was tested using two different media (BHI and TBS) and the cells were harvested 3 hours and 24 hours after induction. A 6xHis-tagged purified EfmRsep was used as a positive control (PC), while a non-induced sample (-) was used as a negative control for all conditions tested. The full-length, original western blots are presented in supplementary Figure S10.

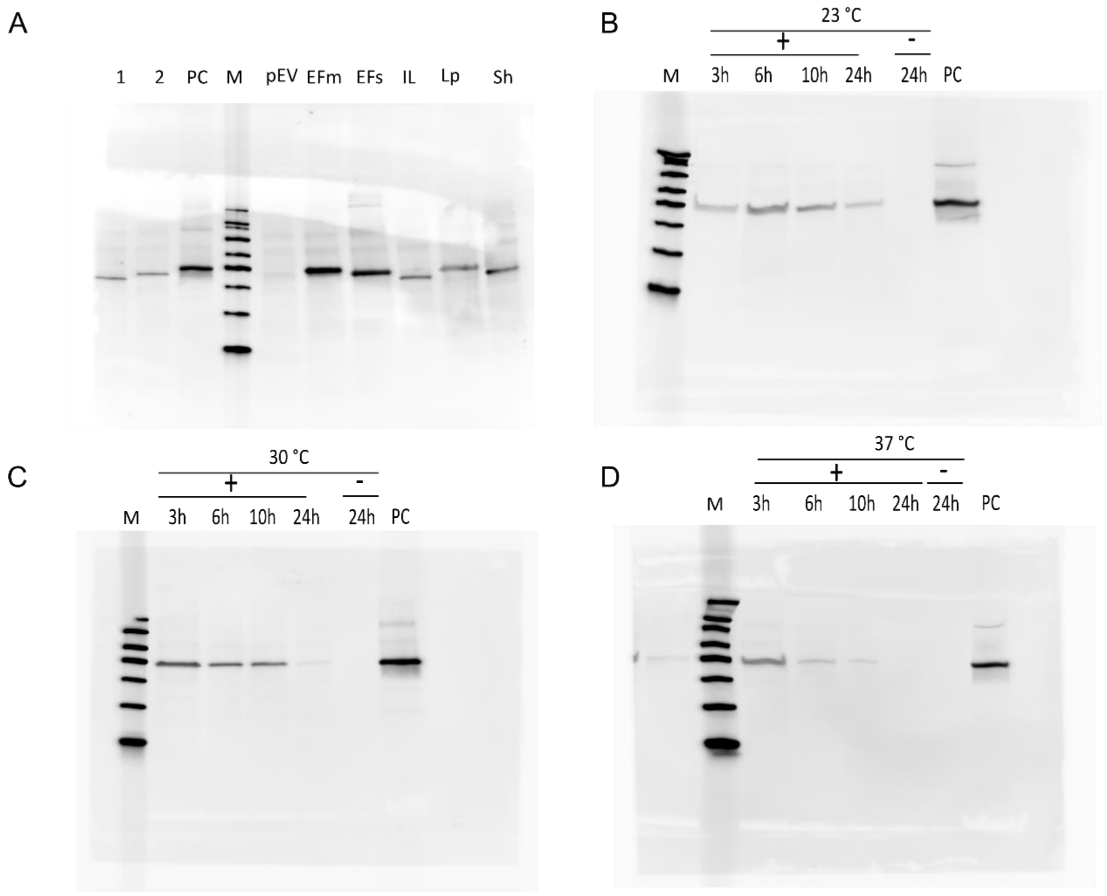


**Figure S6 | Original, full-length western blots applied in Figure 2.** A) Heterologous production of RseP derived from five gram-positive bacteria was evaluated in *L. plantarum* WCFS1 using western blot analysis: *E. faecium* (Efm), *E. faecalis* (Efs), *L. lactis* (Ll), *L. plantarum* (Lp) and *S. haemolyticus* (Sh). Lanes 1 and 2 contain samples unrelated to this article. B-D) Small-scale expression screening of EfmRseP was conducted in 50 ml cultures at three temperatures: 23°C (B), 30°C (C) and 37°C (D) and harvested at various time points after induction: 3-, 6-, 10- and 24 hours. Induced samples are indicated with +, while non-induced samples are marked with -. In all blots: M: Molecular Marker, PC: Positive control.

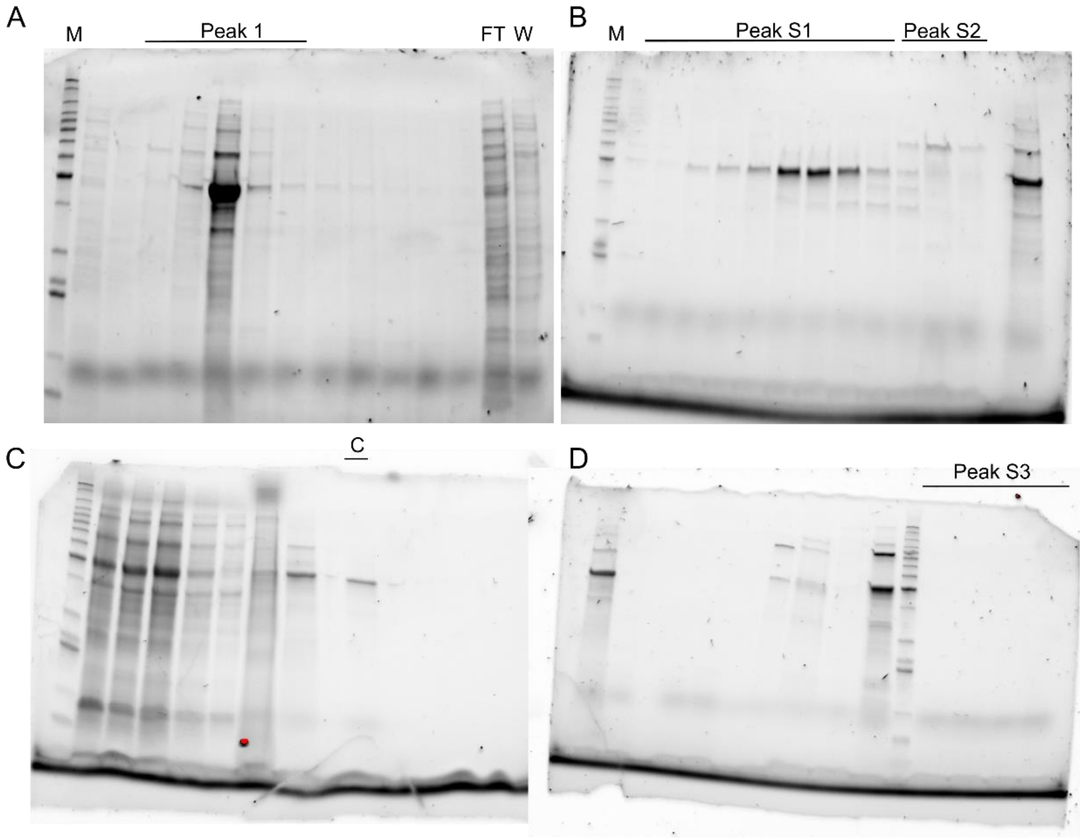


**Figure S7 | Original, full-length SDS-PAGE applied in Figure 4.** A) SDS-PAGE showing analyzed IMAC fractions. B-D) SDS-PAGE showing analyzed SEC fractions. Regions included in Figure 4 are labeled.

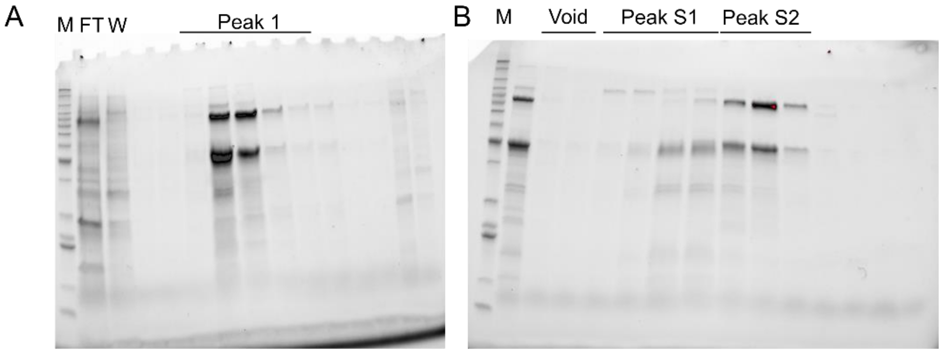


**Figure S8 | Original, full-length SDS-PAGE applied in Figure 5.** A) SDS-PAGE showing fraction from the IMAC chromatography. B) SDS-PAGE showing fraction from SEC. Regions included in Figure 5 is labeled.

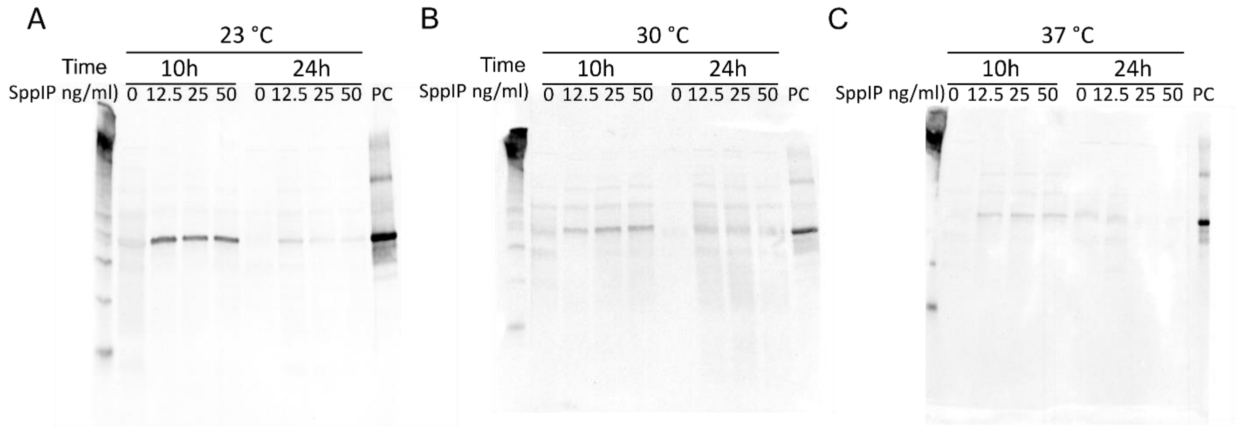


**Figure S9 | Original, full-length western blot applied in Figure S2.** Western blot analysis showing the effect of inducer concentration on pro-longed protein expression (10- and 24 hours post-induction) at 23 °C (A), 30 °C (B) and 37 °C (C).

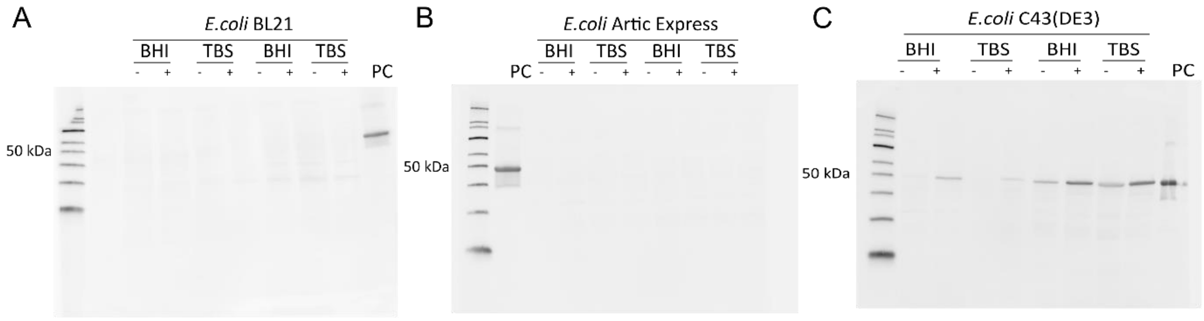


**Figure S10 | Original, full-length western blot applied in Figure S5.** Western blot analysis of protein production in *E. coli* harboring pET22b-EfmRseP. The small-scale expression screen was performed using three strains: *E. coli* Artic Express (A), *E. coli* BL21 Star™ (DE3) (B), and *E. coli* C43(DE3) (C).
